# Unveiling the impact of C15orf48 on non-small cell lung cancer through NF-kappa B signaling

**DOI:** 10.17305/bb.2024.11113

**Published:** 2024-11-22

**Authors:** Wei Wang, Lei Zhang, Ansheng Wang, Xiaohua Wang, Weidong Wu

**Affiliations:** 1 Department of Thoracic Surgery, Guangzhou Red Cross Hospital affiliated to Jinan University, Guangzhou, China; 2 Department of Thoracic Surgery, The First Affiliated Hospital of Bengbu Medical University, Bengbu, China; 3 State Key Laboratory of Respiratory Disease, National Clinical Research Center for Respiratory Disease, Guangzhou, China; 4 Institute of Respiratory Health, First Affiliated Hospital of Guangzhou Medical University, Guangzhou, China

**Keywords:** C15orf48, non-small cell lung cancer, NF-κB signaling pathway, immune cell infiltration

## Abstract

The role of the C15orf48 gene in lung cancer is not well understood. This study aimed to investigate the effect of C15orf48 in non-small cell lung cancer (NSCLC). Bioinformatics analyses were performed using Oncomine, The Cancer Genome Atlas (TCGA), Protein-Protein Interaction (PPI) networks, Gene Ontology (GO), Kyoto Encyclopedia of Genes and Genomes (KEGG), and Gene Set Enrichment Analysis (GSEA). Immunohistochemical staining was used to detect C15orf48 expression in tissue microarrays. Cellular assays, including CCK8, colony formation, wound healing, transwell migration, flow cytometry, and cell adhesion, were conducted to assess cell viability, proliferation, invasion, and apoptosis. A xenograft tumor model was used to examine tumor growth, and Western blotting was used to detect protein expression. C15orf48 expression was significantly upregulated in tumor tissues compared to normal tissues and was associated with poor prognosis. Knockdown of C15orf48 in A549 and H1299 cells reduced proliferation, invasion, and adhesion while increasing apoptosis. C15orf48 knockdown also inhibited tumor growth *in vivo* and was associated with immune cell infiltration. Although C15orf48 expression correlated with the epithelial-mesenchymal transition (EMT) score, no significant differences were observed. GSEA identified the NF-κB signaling pathway as a key pathway involved. Proteins PLAUR, IKBα, IL-1RN, ICAM1, and TMPRSS4 showed decreased expression in the shC15orf48 group compared to the shCtrl group. We concluded that C15orf48 promotes NSCLC progression, potentially through immune cell infiltration and the NF-κB signaling pathway.

## Introduction

Lung cancer is the most common cancer worldwide and the leading cause of cancer-related deaths, with about 1.8 million new cases diagnosed annually [1]. It has a poor prognosis, with over two-thirds of patients developing metastases [2]. Current diagnostic tools, such as chest radiographs and sputum cytology, lack sensitivity for the early detection of non-small cell lung cancer (NSCLC), and tumor markers like CEA, CYFRA 21–1, NSE, and SCCA are not effective for early-stage diagnosis [3]. There is a critical need for more specific, less invasive biomarkers for lung cancer detection and staging [4].

C15orf48 (Chromosome 15 Open Reading Frame 48), also known as NMES1, Coxfa4l3, MISTRV, or MOCCI, is a small mitochondrial protein comprising 83 amino acids [5]. It is located on human chromosome 15 and has gradually attracted the attention of researchers in recent years. Although studies on the function and biological significance of this gene are still ongoing, some preliminary findings have been made. Evidence suggests that this gene may play an important role in several types of cancer. For example, the expression level of C15orf48 is significantly altered in glioma patients, and its overexpression is associated with poor prognosis, suggesting that it may serve as a potential biomarker [6]. In papillary thyroid carcinoma (PTC), C15orf48 has been associated with immune cell infiltration, suggesting a role in the tumor microenvironment [7]. Moreover, C15orf48 has been shown to regulate epithelial cell metabolism via the C15orf48/miR-147 axis, thereby modulating host inflammation and immunity in various conditions including gastrointestinal diseases. These findings highlight the involvement of this gene in cancer progression through mechanisms, such as immune regulation and inflammation [8, 9]. Although previous studies have explored the role of C15orf48 in various cancers and its potential link to immune responses and inflammatory processes, its specific function in NSCLC remains largely unexplored.

Based on the known function of C15orf48 in other cancers and its potential involvement in immune cell infiltration and inflammation, this study aims to investigate the expression and function of C15orf48 in NSCLC, focusing on its role in tumor growth, immune cell infiltration, and related signaling pathways. By addressing these questions, we sought to determine whether C15orf48 could serve as a potential biomarker or therapeutic target for NSCLC.

## Materials and methods

### Analysis of online datasets

The Oncomine database was utilized to analyze the expression of the C15orf48 gene in lung cancer. RNA sequencing (RNA-seq) data and clinical data for lung adenocarcinoma (LUAD) from The Cancer Genome Atlas (TCGA) were acquired and systematically organized, including 59 normal and 541 tumor samples. Differential expression plots for C15orf48 were generated using the “ggplot2” package, and statistical analysis was performed with the Wilcoxon test. Kaplan–Meier (KM) survival analysis was conducted and visualized on the TPM matrix, incorporating relevant clinical information, using the “survival” and “survminer” packages. The data were divided into high and low expression groups based on median gene expression levels. The effectiveness of C15orf48 in distinguishing and diagnosing LUAD patients was assessed relative to the control group within the TCGA-LUAD dataset using the pROC package. An area under the curve (AUC) value approaching 1 indicates a stronger association with the disease.

### Evaluation of immune cell infiltration in LUAD

Using the “CIBERSORT” package, immune infiltration was assessed on the TCGA-LUAD RNA-seq data in TPM format based on the CIBERSORT algorithm, which provided the abundance of 22 immune cell types. Spearman correlation analysis was then performed to determine the relationships between target genes and immune cell types. Additionally, data visualization was conducted for classic pathways. Spearman correlation was also calculated between 79 immune checkpoint genes, as reported in a previous study [10], and the target genes, identifying those with *P* values less than 0.05. Visualizations were created based on these correlation results.

### EMT score

Genes associated with epithelial–mesenchymal transition (EMT) were selected from previous research, including 25 epithelial and 52 mesenchymal marker genes [11, 12]. EMT scores for TCGA-CRC patients were calculated, and Spearman correlation analysis was performed to assess the relationship between these scores and the target gene C15orf48.

### Lung cancer tissue microarray and IHC staining

A human lung cancer tissue array (IWLT-N-96LN91) was purchased from Wuhan Biological Technology Co., Ltd. (Wuhan, China), and included 47 lung cancer tissue samples and adjacent normal tissue samples. Immunohistochemical (IHC) staining was performed to detect C15orf48 protein levels using a primary antibody (Anti-C15orf48 antibody, 1:200 dilution, Abcam, Cambridge, MA, USA) at room temperature for 2 h. Following washing with phosphate-buffered solution (PBS), the sections were incubated with secondary antibodies at room temperature for 1 h. The slides were then stained with diaminobenzidine, counterstained with hematoxylin, dehydrated, and mounted. Each slide was independently reviewed and scored by two observers who were not involved in the experiment.

### Cell lines and cell culture

Human bronchial epithelial cells (BEAS-2B) and human lung cancer cell lines (A549, H1299, PC9, and H23) were purchased from the Shanghai Cell Bank of the Chinese Academy of Sciences. All cells were cultured at 37 ^∘^C in RPMI-1640 medium (Gibco, C11875500BT) supplemented with 10% fetal bovine serum, 100 µg/mL penicillin, and 100 µg/mL streptomycin in a 5% CO_2_ atmosphere. When the cells reached approximately 80% confluence, they were detached using 0.25% trypsin and subcultured.

### RNA interference

The RNA interference assay for siC15orf48 and the negative control siCtrl were obtained from GenePharma Co. (Shanghai, China). Three siC15orf48 sequences siC15orf48-1 were tested: Sense strand 5′-GCUUAUAACAAUCAACCAACATT-3′ and antisense strand 5′-UGUUGGUUGAUUGUUAUAAGCTT-3′; siC15orf48-2: Sense strand 5′-GAAGGAACUCAUUCCCUUG GUTT-3′ and antisense strand 5′-ACCAAGGGAAUGAGUU UCCUUCTT-3′; siC15orf48-3: Sense strand 5′-GGAAACCCAUUG AAGAGUUTT-3′ and antisense strand 5′-GCAGAAAAG GGAAAAAGCUUC-3′. The siCtrl sequence consisted of the sense strand 5′-UUCUCCGAACGUGUCACGUTT-3′ and the antisense strand 5′-AACUCUUCAAUGGGUUUCCAT-3′. The siRNA was transfected into A549 and H1299 cells using RNAiMAX (Invitrogen, CA, USA) according to the manufacturer’s instructions. The expression level of C15orf48 was measured using RT-qPCR.

### Lentiviral infections

Lentiviral particles carrying C15orf48 cDNA and the GFP vector were obtained from GeneChem (Shanghai, China) and used to transfect A549 and H1299 cells. Experiments were performed using the Lentivirus Packaging Kit (GeneChem, Shanghai, China). Specifically, the cell culture medium was replaced with Opti-MEM medium 2 h before transfection. Twenty microliters (20 µL) of GFP Control Plasmid and 25 µL of Lenti-Easy Packaging Mix were mixed well with the corresponding volume of Opti-MEM, a total volume of 1.5 mL, and incubated at room temperature for 5 min. The Lipofectamine 2000 Reagent was gently shaken, and 60 µL of Lipofectamine 2000 was mixed with 1440 µL of Opti-MEM in a total volume of 1.5 mL and incubated for 5 min at room temperature. The diluted packaging system was mixed with the diluted Lipofectamine 2000, by gently inverting the mixture (3 mL total). The mixture was incubated at room temperature for 20 min to form a transfection complex of DNA with the Lipofectamine 2000 dilution. The transfection system mixture was transferred to the 293T cell culture medium, mixed well, and incubated at 37 ^∘^C with 5% CO_2_ in a cell culture incubator. After transfection, the medium was changed at 24 h to remove unbound virus particles and replenished with fresh medium. The cells were incubated for 48–72 h for viral infection and expression. GFP expression was observed using a fluorescence microscope to confirm transfection.

### RNA extraction and RT-qPCR

Total RNA was extracted from the collected tissues and each cell line using TRIZOL reagent (Invitrogen). Next, reverse transcription reactions were performed using SuperScript III First-Strand Synthesis SuperMix for RT-qPCR (Takara). Three replicates of RT-qPCR were performed using the following cycling conditions: 95 ^∘^C for 60 s, 95 ^∘^C for 15 s, and 63 ^∘^C for 25 s for 40 cycles. GAPDH was used as endogenous control and analyzed using method 2^--ΔΔCT^ Primers (Shanghai Bioengineering Co., Ltd.): human GAPDH, forward, 5′-CCATGACAACTTTGGTATCGTGGAA-3′, reverse, 5′-GGCCATCACGCCACA GTTTC-3′; human C15orf48, forward, 5′- AGGAAGGAACTCATTCCCTTGG-3′ and reverse, 5′-TTTTTGAGGTACAGTAGGGTCCA-3′.

### Western blotting

Cells were lysed in RIPA buffer containing a protease inhibitor mixture and phenyl-methanesulfonyl fluoride. Total cellular proteins were extracted using T-PER tissue extraction reagent (Thermo Pierce, USA) with 1% Halt protease and phosphatase inhibitor (Thermo Pierce, USA). The protein concentration was determined using a BCA reagent (Thermo Pierce, USA). Proteins were separated by SDS-PAGE (10% separation gel, 5% concentration gel) and transferred to polyvinylidene difluoride (PVDF) membranes (Millipore, USA). The PVDF membranes were sealed in 5% skim milk and Tris-buffered saline containing 0.1% Tween -20 detergent (TBST) at room temperature for 2 h. The membranes were then incubated with anti-C15orf48 (Invitrogen, USA), IKBα (1:1000, Bioss, Beijing, China), IL-1R1 (1:2000, Bioss, USA), Transmembrane Protease Serine 4 (1:1500, TMPRSS4, Bioss), and PLAUR (1:1000, Abcam, UK). The membranes were washed three times with TBST for 10 min each and then mixed with horseradish peroxidase-labeled goat anti-mouse IgG or goat anti-rabbit IgG secondary antibody (ZsBio, Beijing, China) for 1.5 h. Protein development was performed using ECL Western Blotting reagent (Thermo Pierce, Rockford, IL, USA). The relative expression of target proteins was quantified using Image J (NIH, Bethesda, MD, USA).

### Cell Counting Kit-8 (CCK-8)

First, siCtrl or siC15orf48 was transfected for 24 h on 96-well plates (5 x 10^3^ cells /well). Absorbance was measured at 450 nm with the CCK-8 reagent (Biomiky, China). 10 µL/well of CCK-8 was added at the same time point each day and incubated for a further 2 h. Absorbance at 450 nm was then measured using an automated enzyme marker. CCK-8 was added for five consecutive days for detection.

### Colony formation assay

For the colony formation assay, transfected cells (500 cells per well) were seeded into 6-well plates. After 14 days of incubation, the cells were fixed with methanol and stained with 0.1% crystal violet. The number of colonies was then counted.

### Apoptosis detection

Apoptosis was detected using the Annexin V FITC/PI kit (Beyotime, Shanghai, China). Cells with different treatments were inoculated in 6-well plates, digested, centrifuged at 1300 rpm for 5 min at 4 ^∘^C, and transferred to a new centrifuge tube. Cells were resuspended in ice-cold PBS and centrifuged again at 1300 rpm for 5 min at 4 ^∘^C. After suspension in PBS, 200 µL of 1× binding buffer was added to each tube and mixed well. Next, 5 µL of Annexin V-FITC and 10 µL of propidium iodide (PI) were added, mixed gently, and incubated for 30 min at room temperature in the dark. Cells were then detected using flow cytometry (BD Bioscience, San Jose, CA, USA).

### Cell cycle analysis

Cells were grouped as described previously. A cell cycle assay kit (Beyotime, Shanghai, China) was used to detect the cell cycle. Briefly, cells were fixed in 70% ethanol, digested with 100 µL RNase A for 30 min, stained with 400 µL PI, and then incubated in the dark for 30 min. Cell cycles were measured and analyzed using flow cytometry (BD Bioscience, San Jose, CA, USA).

### Wound healing assay

Wound healing assays were performed by inoculating cells at a density of 5 × 10^5^/well in 12-well plates and incubating them for 24 h to achieve approximately 90% fusion. A scratch line was drawn with the tip of a 10 µL pipette. Cells were rinsed three times with PBS to remove the scratched cells and were cultured in a medium containing 0.5% fetal bovine serum. Cells were incubated at 37 ^∘^C for 6 h and photographed with a light microscope (Olympus Corporation, Tokyo, Japan) for 24 h. PBS was used as a control.

### Transwell migration and invasion assay

Cell migration and invasion were assessed using transwell chambers (Millipore, Bedford, MA, USA) placed in 24-well plates. For the invasion assay, the transwell chambers were coated with Matrigel. A total of 200 µL of serum-free medium containing 5×10^5^ cells was added to the upper chamber, while 750 µL of complete medium with fetal bovine serum was added to the lower chamber as a chemoattractant. After incubation, non-invading cells on the upper side of the filter were removed with a cotton swab. Cells that had migrated or invaded to the lower side of the membrane were fixed with methanol and stained with crystal violet solution. Migrated or invaded cells were observed and photographed using an Olympus light microscope (Olympus Corporation, Tokyo, Japan) at 200× magnification. Images were taken from three random fields for each of the three replicate wells, and the number of migrated or invaded cells was counted.

### Cell adhesion assay

96-well plates were precoated with fibronectin (Sigma-Aldrich, Louis, MO, USA) and blocked with 1% bovine serum albumin (BSA; Sigma-Aldrich) for 2 h at 37 ^∘^C. Cells (3×10^4^) in serum-free medium were seeded into the plates. After a 2-h incubation, adherent cells were fixed with 4% paraformaldehyde and stained with 0.5% crystal violet (Sangon Biotech). The dye was dissolved using sodium dodecyl sulfate (Amresco, Solon, OH, USA), and the absorbance at 570 nm was measured using a microplate reader.

### Xenograft tumor model

All animal studies were conducted in accordance with protocols approved by the Ethics Committee of Bengbu Medical College, People’s Republic of China. Animals were housed according to animal care guidelines and had free access to food and water under 12-h light/dark conditions. A total of 5×10^6^ shC15orf48 and shCtrl lentivirus-infected H1299 cells for 48 h were injected subcutaneously into the right side of female BALB/C mice (six weeks old). Tumor growth was monitored weekly using calipers, and in the fifth week after injection, the mice were executed and tumors were collected for analysis. Tumor volume was calculated as V ═ (a×b^2^)/2, where is the long axis and b is the short axis. All animal work was approved by the Animal Care Committee of the Bengal Medical College in accordance with the guidelines of the Institutional Animal Care and Use Committee (Approve number: 2023436).

### Ethical statement

The experimental protocols were approved by the Ethics Committee of the First Affiliated Hospital of Bengbu Medical University (approval number: 2023436).

### Statistical analysis

Statistical analysis was performed using GraphPad Prism 8.0. Differences between experimental and control groups were assessed using Student’s *t*-test. *P* values of 0.05 or less were considered statistically significant in two-tailed tests. Data are shown as the mean ± SD from three independent experiments.

## Results

### High expression of C15orf48 in NSCLC was associated with poor patient prognosis

**Figure 1.**
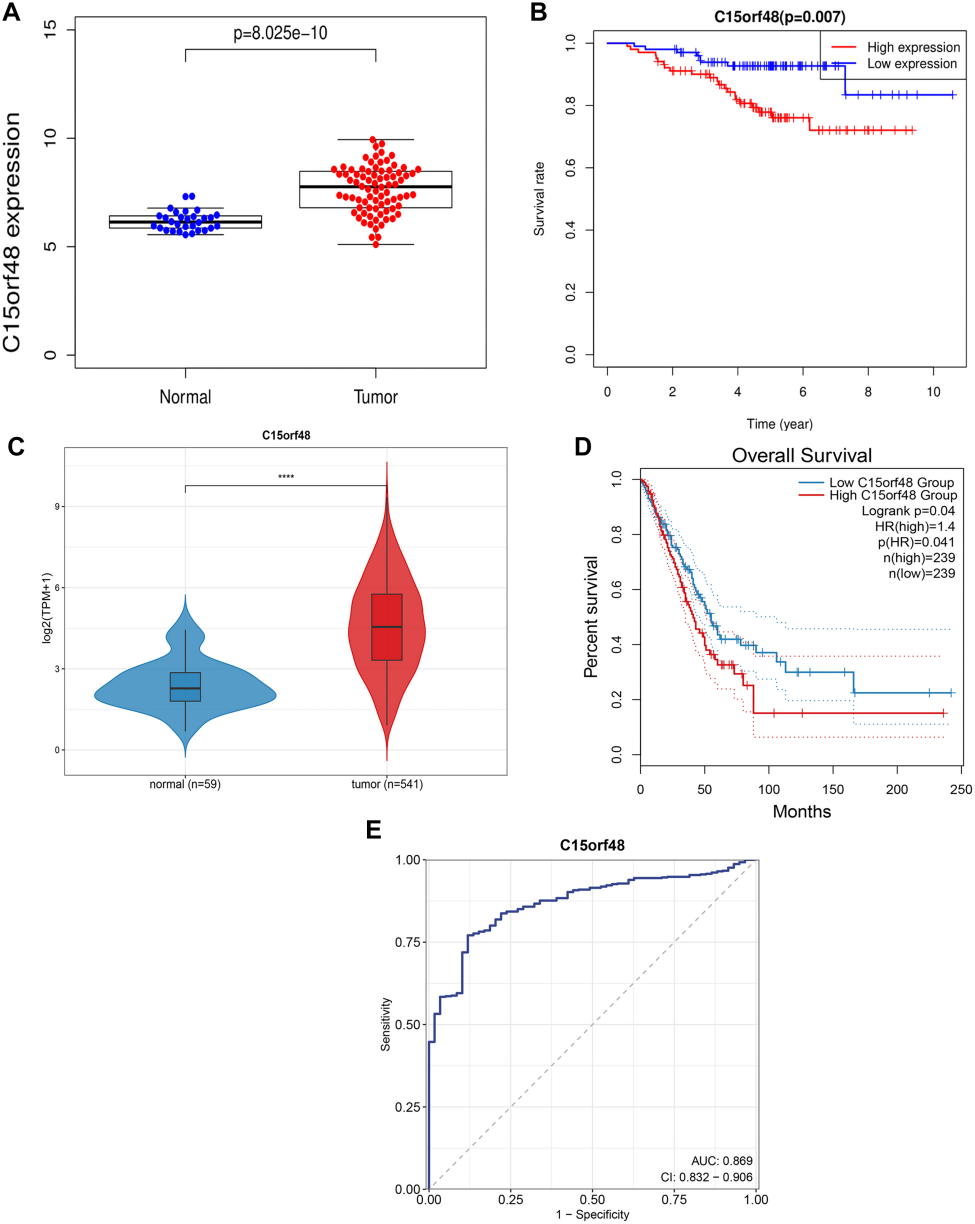
**Elevated expression of C15orf48 in NSCLC.** (A) Expression of C15orf48 in NSCLC tissues and normal adjacent tissues from the Oncomine database; (B) Patients with NSCLC and high C15orf48 expression have a poor prognosis; (C) Expression of C15orf48 in NSCLC tissues and normal adjacent tissues from the TCGA database; (D) Patients with NSCLC and high C15orf48 expression have a poor prognosis; (E) ROC result revealed that high C15orf48 expression has a good predictive value. ****P < 0.0001 compared to the normal group. NSCLC: Non-small cell lung cancer; TCGA: The Cancer Genome Atlas.

Lung cancer expression profile data from the Oncomine database showed that C15orf48 expression was upregulated in lung cancer (Figure 1A). High expression of C15orf48 was associated with poor prognosis in NSCLC patients (Figure 1B). TCGA database results also demonstrated the high expression of C15orf48 in the tumor group compared to the normal group (Figure 1C). The HR value indicated a high risk of C15orf48 expression with low OS (Figure 1D). Furthermore, we performed an ROC analysis of C15orf48 in 20 cancers using public datasets. The results showed that C15orf48 exhibited significant predictive value in multiple cancer types. The AUC value for NSCLC was 0.869, indicating that the expression of C15orf48 has a good predictive value (Figure 1E and Figure S1).

### C15orf48 was highly expressed in lung cancer tissue microarrays and NSCLC cell lines

**Figure 2.**
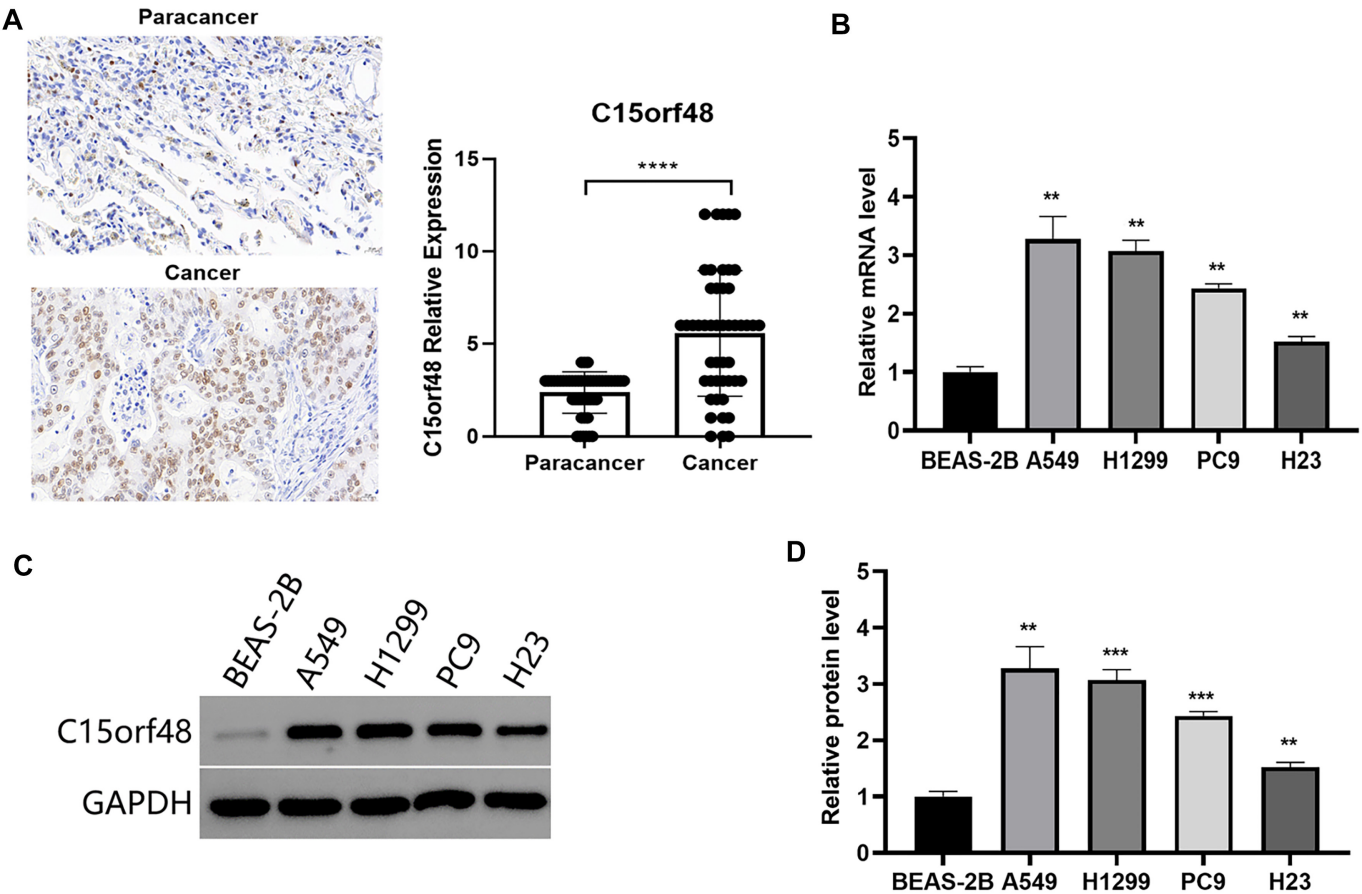
**Expression of C15orf48 in lung cancer tissue microarrayand NSCLC cell lines.** (A) Representative images of C15orf48 immunostaining in paracancerous and cancer tissues (scale bar: 20 µm). **** *P* < 0.0001. (B) Relative levels of C15orf48 mRNA in BEAS-2B and NSCLC cell lines. ** *P* < 0.01 compared to BEAS-2B cells. (C and D) Western blot analysis was used to detect the relative expression levels of C15orf48 protein in BEAS-2B and NSCLC cell lines. ** Compared to BEAS-2B cells, *P* < 0.01. NSCLC: Non-small cell lung cancer.

IHC was performed on LUAD and adjacent tissues. The results showed that C15orf48 was highly expressed in cancer tissue compared to adjacent cancer (Figure 2A). To select a suitable cell model for investigating the biological function of C15orf48, we examined the relative expression levels of the C15orf48 gene in BEAS-2B cells and four lung cancer cell lines. The results showed that the mRNA and protein expression levels of C15orf48 were higher in A549, H1299, PC9, and H23 cells compared to the BEAS-2B cells (Figure 2B--2D, *P* < 0.01). Among these, A549 and H1299 had higher expression levels than PC9 and H23 cells, therefore, A549 and H1299 were selected for subsequent functional experiments.

**Figure 3.**
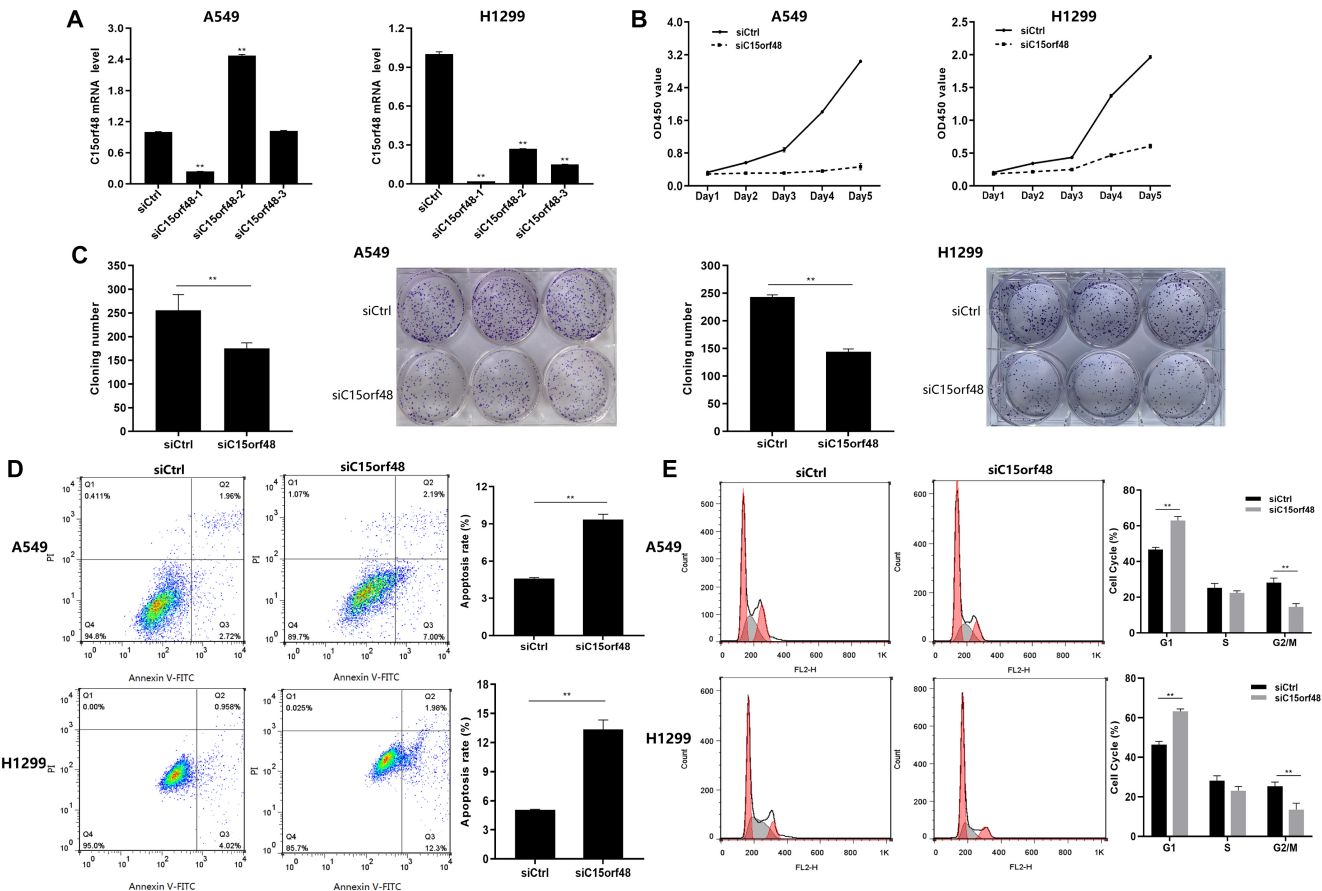
**C15orf48 knockdown inhibited proliferation and promoted apoptosis in NSCLC cells.** (A) RT-qPCR to determine the relative C15orf48 mRNA expression levels in A549 and H1299 cells transfected with siCtrl and siC15orf48; (B) Cell viability in A549 and H1299 cells was assessed using the CCK-8 assay; (C) Retarded growth of A549 and H1299 cells in the plates was measured using a colony formation assay; (D) AnnexinV-FITC/PI double-staining flow cytometry showed increased apoptosis in the siC15orf48 group compared to the siCtrl group; (E) The percentage of cells in the G1 phase, S phase, and G2/M phase were analyzed by flow cytometry. *P < 0.05, ** *P* < 0.01 compared to the siCtrl group. NSCLC: Non-small cell lung cancer.

### C15orf48 knockdown inhibits NSCLC cell proliferation and promotes apoptosis

First, we knocked down the expression of the C15orf48 gene in A549 and H1299 cells using siC15orf48 transfection. Compared with the siCtrl group, the knockdown effect of siC15orf48-1 was the most significant (Figure 3A, *P* < 0.01). Thus, siC15orf48-1 was used for subsequent experiments. The results of the CCK-8 and colony formation assays showed that cell proliferation and colony formation capacities were significantly decreased in the siC15orf48 group compared to the siCtrl group (Figure 3B and 3C, *P* < 0.01). Furthermore, flow cytometry results indicated that cell apoptosis was significantly increased in the siC15orf48 group compared to the siCtrl group (Figure 3D, *P* < 0.01). This finding suggests that C15orf48 may play a role in inhibiting apoptosis in lung cancer cells. Cells in the siC15orf48 group showed a significant increase in the G1 phase and a significant decrease in the proportion of cells in the G2/M phase, while the number of cells in the S phase did not change significantly, indicating that C15orf48 promotes cell cycle progression, thereby facilitating tumor growth (Figure 3E, *P* < 0.01). These findings suggest that the mechanism of action of C15orf48 in lung cancer may involve multiple levels of regulation, including cell cycle progression, cell proliferation, apoptosis, and cell–cell interactions. By inhibiting the expression of C15orf48, the proliferation and colony formation ability of lung cancer cells can be significantly suppressed, and cell apoptosis can be promoted, thereby inhibiting the progression of lung cancer.

### C15orf48 knockdown inhibits migration, invasion, and adhesion of NSCLC cells

**Figure 4.**
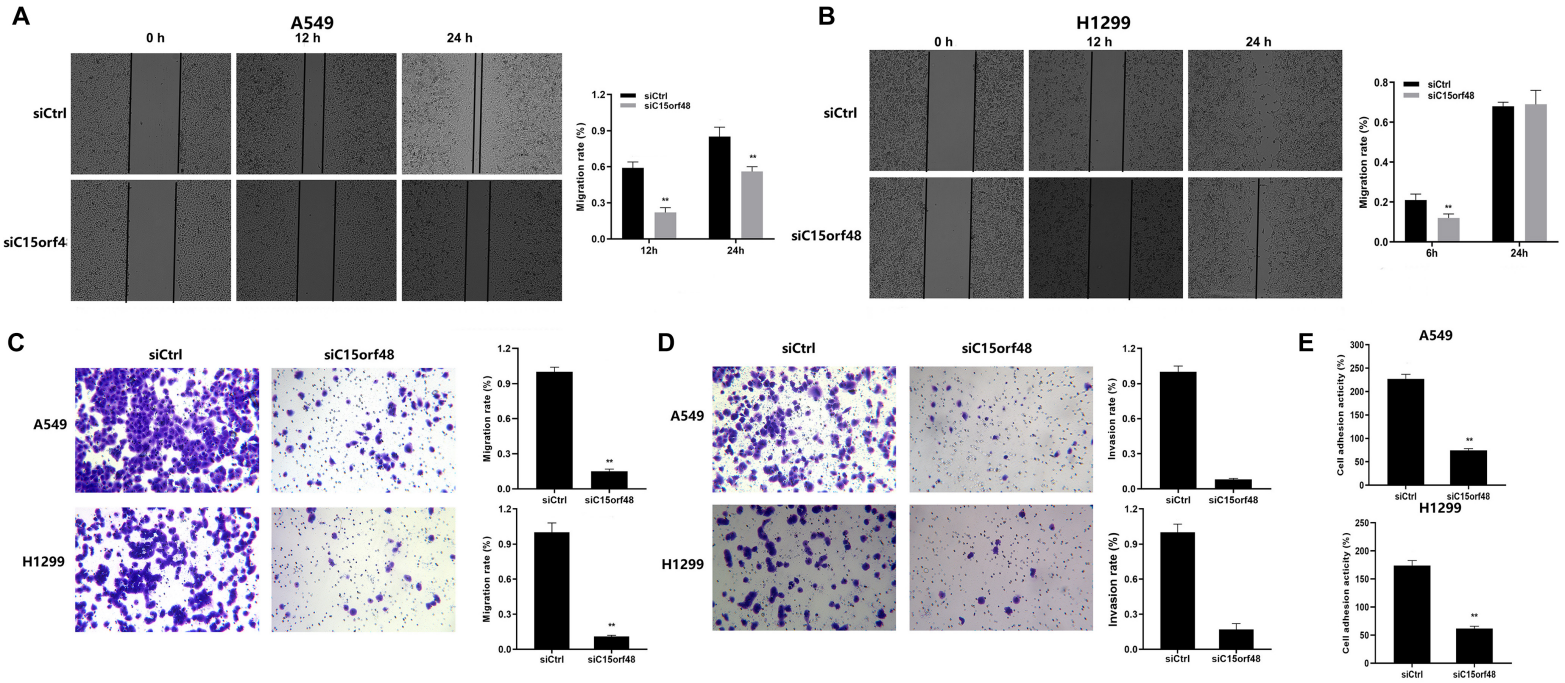
**C15orf48 knockdown inhibits migration, invasion, and adhesion of NSCLC cells.** (A and B) Scratch assay showing a reduced 24-h migration rate of A549 and H1299 cells in the siC15orf48 group compared to the siCtrl group; (C and D) Transwell assays showed that the downregulation of C15orf48 significantly reduced the migration and invasion abilities of A549 and H1299 cells; (E) Adhesion assay showing reduced cell adhesion activity following the downregulation of C15orf48. *P < 0.05, **P < 0.01 compared to the siCtrl group. NSCLC: Non-small cell lung cancer.

To better understand the role of C15orf48 in NSCLC progression, we investigated several key characteristics associated with cancer metastasis, including cell migration, invasion, and adhesion. These processes are critical for the spread of cancer cells from the primary tumor to distant sites, making them important indicators of metastatic potential. We found that the migration and invasion abilities of A549 and H1299 cells were significantly decreased in the siC15orf48 group compared to the siCtrl group, as shown in Figure 4A–4D. The reduction in invasion through Matrigel further supports the role of C15orf48 in promoting NSCLC cell invasiveness, indicating that this gene facilitates the ability of cancer cells to penetrate surrounding tissues—a key step in metastasis. In addition, cell adhesion assays revealed that the adhesion activity of A549 and H1299 cells was markedly decreased in the siC15orf48 group compared to the siCtrl group (*P* < 0.05) (Figure 4E). Reduced adhesion capacity suggests that C15orf48 is involved in enhancing the interaction between cancer cells and the extracellular matrix (ECM), which is essential for cells to maintain their position and initiate metastasis. The weakened adhesion likely facilitates cell detachment from the primary tumor, further promoting metastasis.

In summary, these findings strongly indicate that C15orf48 plays a pivotal role in the metastatic behavior of NSCLC cells by enhancing migration, invasion, and adhesion. The reduction in these key metastatic traits following C15orf48 knockdown suggests that this gene may be a promising target for therapies aimed at limiting NSCLC progression and metastasis.

### C15orf48 knockdown inhibits the growth of tumors *in vivo*

**Figure 5.**
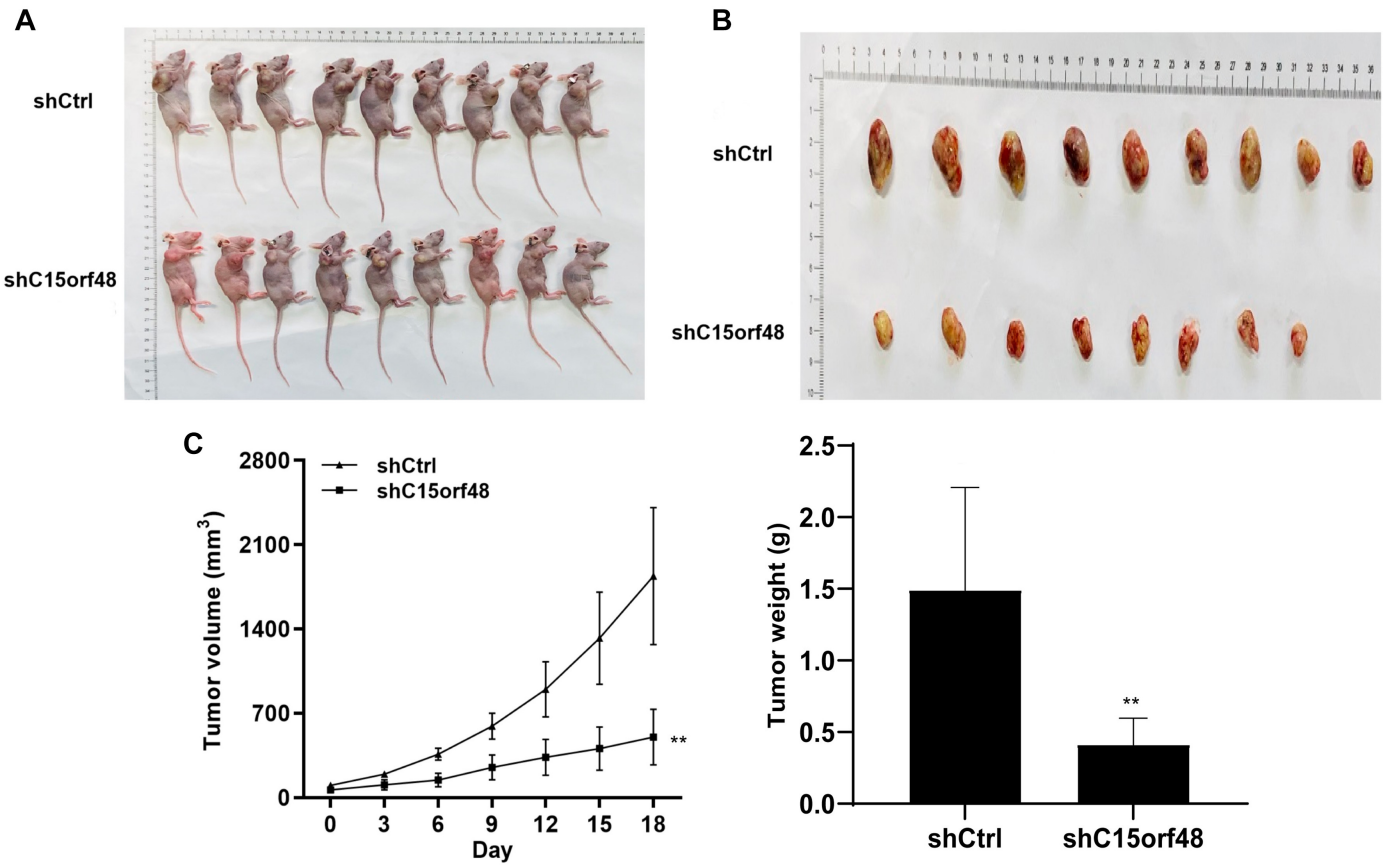
**Nude mice and tumors after knockdown of C15orf48 by injection of H1299 cells.** (A) Image of nude mice; (B) Tumor images; (C) Tumor volume and weight were monitored at the indicated times. ** *P* < 0.01 compared to the shCtrl group.

We used a nude mouse xenograft model to investigate the role of C15orf48 knockdown. The results revealed that the tumor volume and weight were significantly decreased in the shC15orf48 group compared to the shCtrl group (Figure 5A–5C). According to Figure 5B, C15orf48 knockdown exhibited impaired tumorigenicity, as evidenced by one mouse failing to form tumors.

### The underlying mechanism of C15orf48 was related to NF-kappa B signaling pathway-related proteins

**Figure 6.**
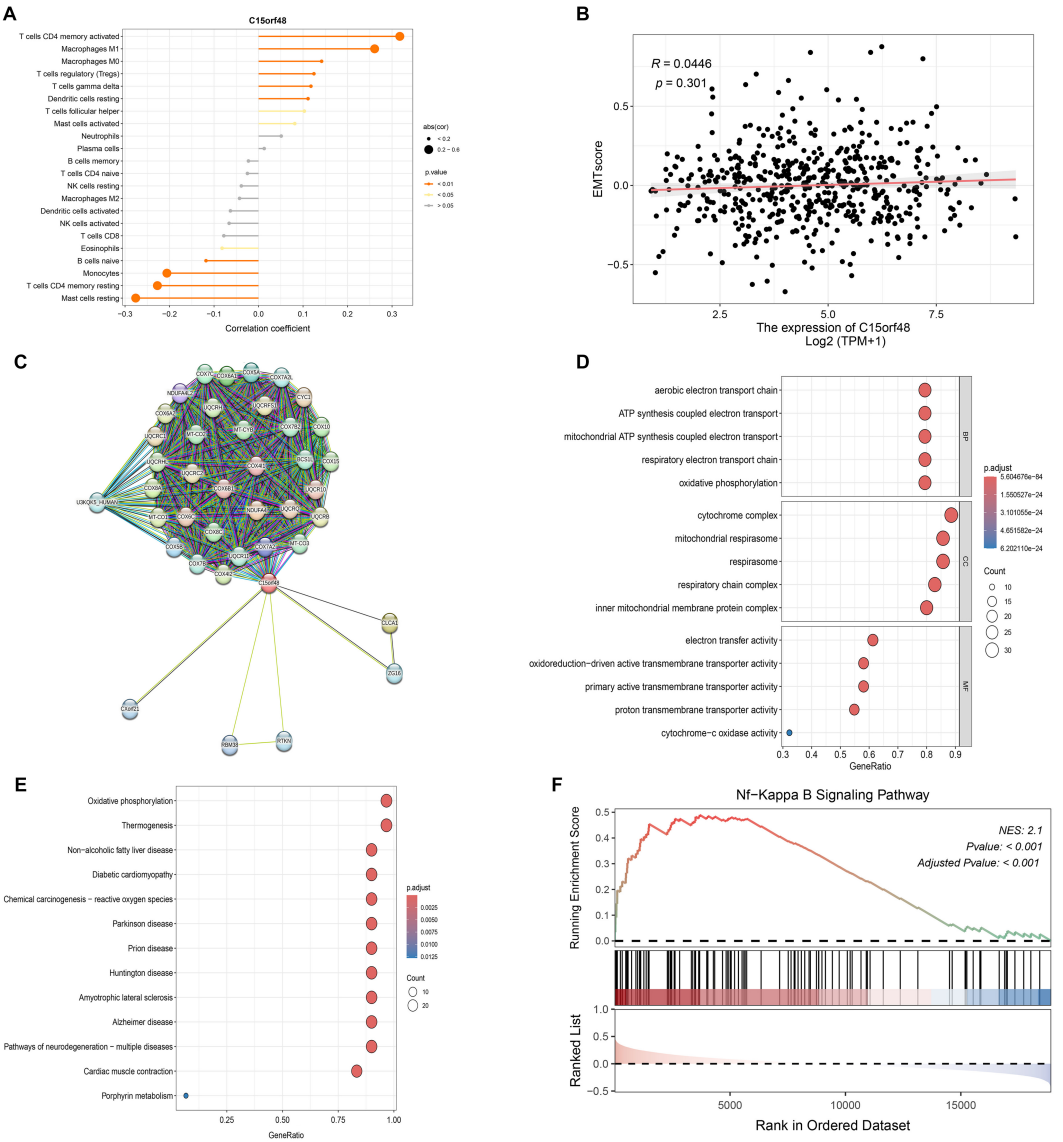
**Molecular Interactions of C15orf48 in NSCLC.** (A) Relationship between the expression of C15orf48 and immune cells; (B) EMT score results from TCGA RNA-seq data; (C) PPI analysis showing the correlation of C15orf48 with other 40 proteins. GO (D) and KEGG (E) functional enrichment analyses were performed on the interacting genes; (F) GSEA analysis results for the NF-κB signaling pathway. NSCLC: Non-small cell lung cancer; EMT: Epithelial–mesenchymal transition; PPI: Protein–protein interaction; GO: Gene ontology; KEGG: Kyoto Encyclopedia of Genes and Genomes; GSEA: Gene Set Enrichment Analysis.

We first analyzed the correlation of C15orf48 with immune cells and EMT scores. The results indicated that C15orf48 was positively correlated with activated CD4 memory T cells, M0 and M1 macrophages, regulatory T cells (Tregs), gamma delta T cells, and resting dendritic cells. Conversely, C15orf48 was negatively correlated with naive B cells, monocytes, resting CD4 memory T cells, and resting mast cells (Figure 6A). Additionally, C15orf48 expression was positively correlated with the EMT score, although the differences were not statistically significant (Figure 6B, *P* > 0.05).

Next, we conducted a PPI (protein–protein interaction) analysis using the STRING database (version 12.0) based on the target gene C15orf48. We retrieved the top 50 proteins with the highest interaction scores with C15orf48, setting the confidence threshold to the default value of 0.4, while keeping all other parameters at their default settings. The results identified 40 C15orf48-related genes (Figure 6C). GO and KEGG functional enrichment analyses were performed on these interacting genes. GO enrichment analysis revealed that the top five enriched biological processes included the aerobic electron transport chain, ATP synthesis coupled to electron transport, mitochondrial ATP synthesis coupled to electron transport, respiratory electron transport chain, and oxidative phosphorylation. In terms of cellular components, enrichment was observed in the cytochrome complex, mitochondrial respirasome, respiratory chain complex, and inner mitochondrial membrane protein complex. For molecular functions, the top enrichments included electron transfer activity, oxidoreduction-driven active transmembrane transporter activity, primary active transmembrane transporter activity, proton transmembrane transporter activity, and cytochrome-c oxidase activity (Figure 6D).

**Figure 7.**
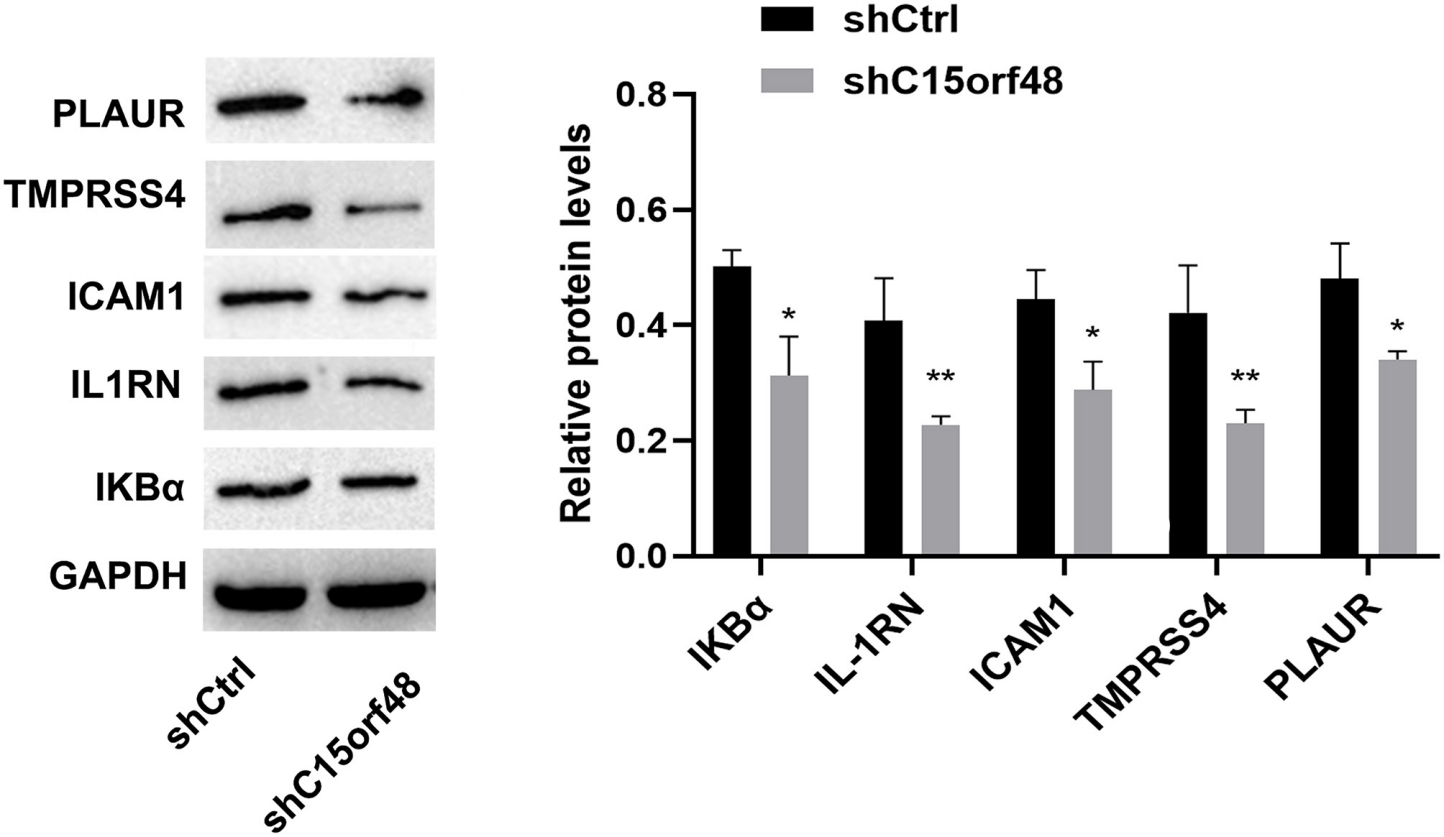
**Expression of NF-κB signaling pathway-related proteins.** Western blot assay results showed decreased expression levels of PLAUR, IKBα, IL-1RN, ICAM1, and TMPRSS4 proteins. Image J software was used for grayscale analysis. * *P* < 0.05 compared to the shCtrl group.

**Figure 8.**
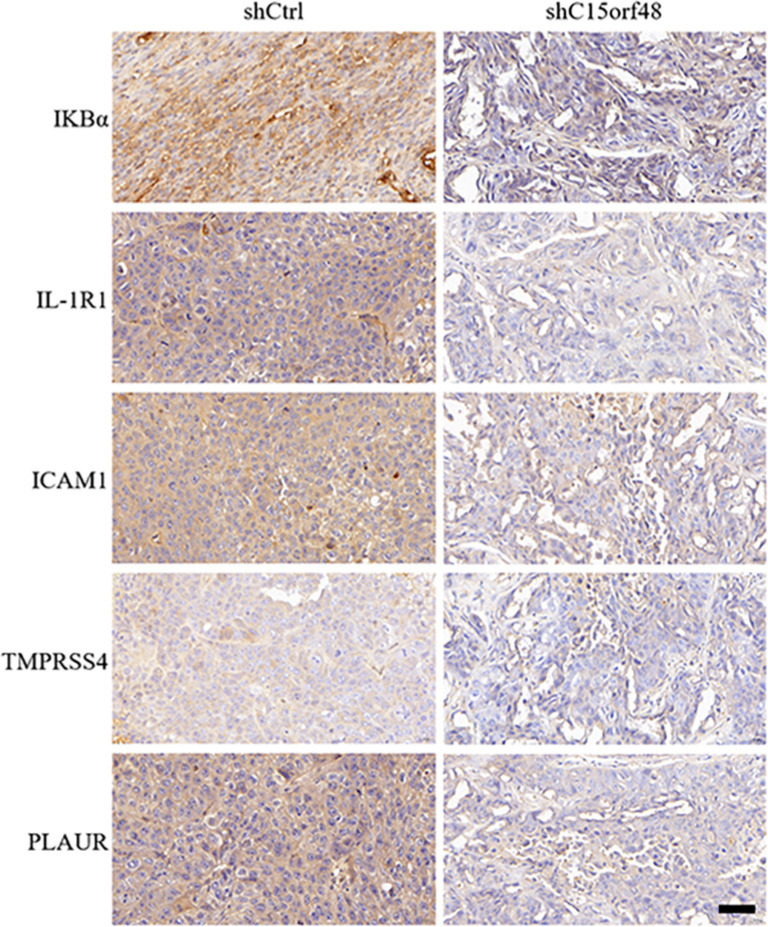
**Expression of NF-κB signaling pathway-related proteins.** IHC assay results showed decreased expression levels of PLAUR, IKBα, IL-1RN, ICAM1, and TMPRSS4 proteins. Scale bar: 50 µm. IHC: Immunohistochemical.

The KEGG functional enrichment analysis indicated that these genes were primarily involved in oxidative phosphorylation, thermogenesis, non-alcoholic fatty liver disease, diabetic cardiomyopathy, chemical carcinogenesis (reactive oxygen species), Parkinson’s disease, prion disease, Huntington’s disease, amyotrophic lateral sclerosis, Alzheimer’s disease, pathways of neurodegeneration (multiple diseases), cardiac muscle contraction, and porphyrin metabolism (Figure 6E). In our KEGG and GO enrichment analyses, although multiple pathways were identified, we focused on the NF-κB signaling pathway because it plays a key role in regulating immune responses and studies have shown that C15orf48 is involved in disease development through the NF-κB signaling pathway. GSEA analysis verified the enriched pathways and confirmed the NF-κB signaling pathway (Figure 6F). The expression levels of PLAUR, IKBα, IL-1RN, ICAM1, and TMPRSS4 proteins were significantly reduced in the shC15orf48 group compared to the shCtrl group (Figures 7 and 8).

## Discussion

Cancer treatment has shifted from broad cytotoxic therapies to targeting specific molecular subtypes [13]. With over one-third of lung cancer patients presenting with advanced disease at diagnosis, systemic therapies—chemotherapy, targeted therapy, and immunotherapy—are essential. However, few nursing home residents with advanced NSCLC receive these treatments [14]. Identifying novel biomarkers and therapeutic targets is critical for improving NSCLC diagnosis and treatment.

C15orf48, initially identified in human esophageal squamous cell carcinoma, is highly expressed in the colon, small intestine, esophagus, and other normal tissues [6, 15]. Our analysis using the Oncomine and TCGA databases revealed that C15orf48 is overexpressed in lung cancer and correlates with poor prognosis. The TCGA database analysis showed an AUC value of 0.869, indicating strong predictive value for C15orf48 expression. Li et al. reported elevated C15orf48 protein levels across multiple cancers, including lung cancer [6, 16]. Previous studies demonstrated that C15orf48 can differentiate squamous cell carcinoma from pseudoepitheliomatous hyperplasia, suggesting its potential utility in challenging diagnostic cases [17]. Consistent with previous findings, our detection of C15orf48 in a lung cancer tissue microarray confirmed its high expression in tumor tissues, implicating its role in lung cancer progression. Additionally, C15orf48 expression was significantly higher in A549, H1299, PC9, and H23 cells compared to BEAS-2B cells, supporting our findings from Oncomine, TCGA, and tissue microarray analyses.

We performed siRNA-mediated knockdown of C15orf48 in A549 and H1299 cells, which exhibit higher C15orf48 expression compared to PC9 and H23 cells. Various assays, including CCK8, colony formation, wound scratch, transwell, and flow cytometry, demonstrated that C15orf48 knockdown inhibited cell viability, proliferation, migration, and invasion, confirming its role in NSCLC progression. This finding aligns with previous studies showing that C15orf48 knockdown also reduced proliferation and migration in thyroid cancer cells [6]. Additionally, *in vivo* experiments using a tumor mouse model showed that C15orf48 knockdown significantly suppressed tumor growth. These findings underscore the biological significance of C15orf48 in lung cancer, as its expression appears to facilitate key processes such as cell proliferation and metastasis, which are critical for tumor progression.

Recent studies have identified Nmes1 (C15orf48) as a novel regulator of mucosal response and intestinal healing [18]. C15orf48 is involved in substituting the mitochondrial cytochrome c oxidase subunit NDUFA4, a conserved response to inflammatory signals with implications for immune-related pathologies [8]. C15orf48 was also found to be significantly associated with macrophage immune infiltration and immune checkpoints in thyroid cancer, suggesting its potential as a biomarker for PTC [6]. The ECM plays a crucial role in tissue homeostasis, and its abnormalities can drive cancer progression and affect therapeutic efficacy [19, 20]. We hypothesized that C15orf48’s role in NSCLC relates to immune cell infiltration and EMT scores. Our findings show that C15orf48 positively correlates with activated CD4 memory T cells, M1 and M0 macrophages, Tregs, gamma delta T cells, and resting dendritic cells, but negatively correlates with naïve B cells, monocytes, resting CD4 memory T cells, and resting mast cells, indicating its involvement in a complex immune environment. Although C15orf48 expression was positively correlated with EMT scores, the difference was not significant. Further analyses, including PPI, GO, KEGG, and GSEA, identified the NF-κB signaling pathway as enriched. Previous research has shown that C15orf48 affects NF-κB signaling and inflammatory responses [9]. Thus, C15orf48’s impact on NSCLC is likely mediated through the NF-κB pathway.

PLAUR regulates plasminogen activation into plasmin, which cleaves ECMproteins and influences cell migration and invasion [21]. IL1RN encodes the IL-1 receptor antagonist (IL1RA), which competitively binds to IL-1 receptors and inhibits the pro-inflammatory effects of IL-1 [22]. ICAM1, a key molecule in cell adhesion and immune cell migration, is upregulated by NF-κB binding to its promoter in cancer cells [23]. TMPRSS4 is a serine protease involved in ECM remodeling and cellular signaling [24]. Our study found that C15orf48 knockdown led to decreased expression of PLAUR, IKBα, IL-1RN, ICAM1, and TMPRSS4, confirming C15orf48’s involvement in the NF-κB signaling pathway and immune response. These molecules are important for cancer cell adhesion, immune signaling, and inflammation. Hence, combining C15orf48 inhibition with NF-κB inhibitors might result in a dual pathway blockade, offering a more effective therapeutic approach for NSCLC patients resistant to traditional therapies.

## Conclusion

In summary, C15orf48 is highly expressed in NSCLC. Its knockdown reduces NSCLC cell viability, metastasis, and adhesion while inhibiting tumor growth *in vivo*. C15orf48 appears to suppress NSCLC progression via the NF-κB signaling pathway. This suggests that C15orf48 may serve as an emerging target and enhance efficacy when combinated with existing targeted therapies, such as EGFR inhibitors. Furthermore, inhibiting C15orf48 may positively impact the efficacy of immunotherapy, which deserves further exploration in future studies.

## Supplemental data

**Figure S1.**
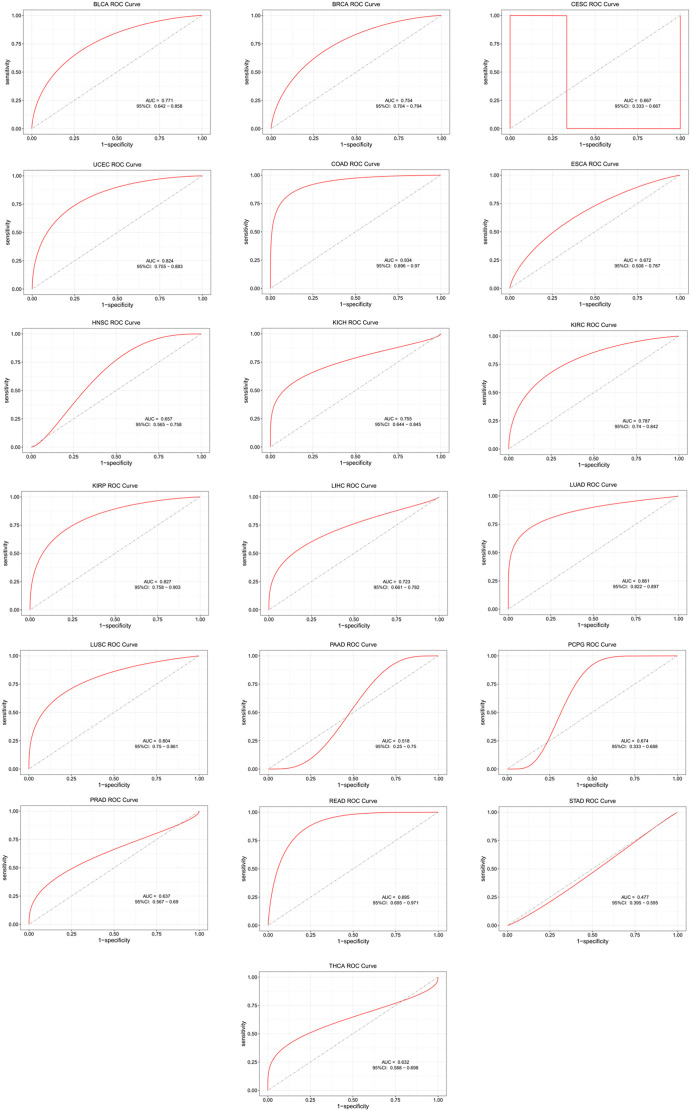
**ROC analysis of the predictive value of C15orf48 in different cancers.** The predictive value of C15orf48 in BLCA, BRCA, CESC, UCEC, COAD, ESCA, HNSC, KICH, KIRC, KIRP, LIHC, LUAD, LUSC, PAAD, PCPG, PRAD, READ, STAD, THCA.

## Data Availability

The data used to support the findings of this study are available from the corresponding author upon request.
